# The role of BRCA status on prognosis in patients with triple-negative breast cancer

**DOI:** 10.18632/oncotarget.19895

**Published:** 2017-08-03

**Authors:** Yuxin Xie, Qiheng Gou, Qianqian Wang, Xiaorong Zhong, Hong Zheng

**Affiliations:** ^1^ Cancer Center, West China Hospital, Sichuan University, Chengdu, Sichuan 610041, P. R. China

**Keywords:** BRCA, triple-negative breast cancer, prognosis, meta-analysis

## Abstract

Studies have showed that dysfunction in the breast cancer susceptibility gene (BRCA) is associated with triple-negative breast cancer (TNBC); however, its effect on patient survival remains controversial. We investigated the distribution of BRCA1/2 mutations in unselected Chinese patients with TNBC and explored their roles in prognosis. Then a systematic review and meta-analysis were performed to evaluate the prognostic role of BRCA dysfunction, including BRCA1/2 germline/somatic mutations, BRCA1 promoter methylation, and low BRCA1 protein expression in TNBC patients. Pooled hazard ratios with 95% confidence intervals were estimated to determine the association between BRCA dysfunction and survival. Our results showed a high frequency of BRCA1/2 mutations, especially germline BRCA1 variants, were associated with bilateral breast cancer. Although no correlations were found between BRCA1/2 mutations and recurrence-free survival (RFS) or overall survival (OS). In the meta-analysis, patients with BRCA1 promoter methylation showed poor OS. However, there was a favorable impact on disease free survival (DFS) for TNBC patients with BRCA1 promoter methylation when received adjuvant-chemotherapy. In conclusion, BRCA1/2 mutations were associated with bilateral breast cancer and BRCA1 promoter methylation may have a prognostic effect on TNBC.

## INTRODUCTION

Breast cancer is a highly heterogeneous disease. Different subtypes show different biological behaviors, therapeutic responses, and clinical outcomes [1]. TNBC is defined by its lack of estrogen receptor (ER), progesterone receptor (PR), and human epidermal growth factor receptor 2 (HER2) expression; and accounts for 10–20% of all breast cancers [2, 3]. Importantly, it is characterized by aggressive clinical behavior and lack of recognized molecular targets for therapy, leading to a poorer prognosis than other breast cancer subtypes [4, 5].

The TN phenotype is the most common histological subtype observed in patients with BRCA1/2 mutations or ‘BRCAness’ breast cancer [4, 6]. ‘BRCAness’ breast cancers are sporadic breast cancers that share the same phenotype or traits with BRCA1/2 mutation tumors [7]. In fact, approximately, 70% of breast cancers with BRCA1 germline mutations are the TN subtype [8]. For TNBC, about 20% patients harbor a BRCA1 mutation [9]. There are some similarities between BRCA1 mutant and TNBC, including the morphological features and immunohistochemical profile. For the morphological features, BRCA1 tumors and TNBC are characterized by high histological grade, atypical medullary features, high proliferation indices, prominent lymphocytic infiltrate and high pushing margins. At the immunohistochemical level, a lot of BRCA1 mutation cancers also show ER/PR-negative, HER2-negative, EGFR overexpression, TP53 mutations and so on [4] [10, 11]. These similarities may have crucial implications for clinical management and prognosis prediction of these cancers. Numerous studies have suggested that BRCA mutation cancer and TNBC show an increased sensitivity to DNA-damaging agents, such as platinum compounds and poly (ADP ribose) polymerase (PARP) inhibitors [12].

BRCA1/2 dysfunctions, including BRCA1/2 mutations, promoter methylation, and protein down-expression, are alternative mechanisms that impair BRCA1/2 function and likely contribute to ‘BRCAness’ genotypes [4, 13]. These abnormality could cause a deficiency in homologous recombination (HR)-mediated DNA double-strand breaks (DSBs) repair. Cells that lack BRCA1/2 to repair these lesions, could tend to more error-prone mechanisms, resulting in an increasing risk to breast cancers [12]. Thus, the role of BRCA1 and BRCA2 mutations in the pathogenesis of breast cancer led us to hypothesize that patients with these mutations might have a worse prognosis than non-carriers. Indeed, studies have demonstrated that BRCA1 mutation decreases short-term and long-term survival; BRCA2 mutation does not increase or decrease either short-term or long-term survival due to the different carcinogenic pathways of BRCA1/2 [14]. Moreover, BRCA1 methylation is also associated with poor survival in breast cancer patients [15]. However, some studies showed that in the TNBC subtype, patients with BRCA1/2 mutations are likely to have a similar or worse survival than non-carriers [16–20]. Thus, these results have been inconsistent. There is an urgent need to accurately determine the prognostic role of BRCA status in patients with TNBC.

## RESULTS

### Prevalence and characteristics of TNBC patients with BRCA1/2 gene mutations

Deleterious BRCA1/2 gene mutations were identified in 15 patients, with an overall mutation frequency of 21.4% (15/70). Of these patients, 14 (93.3%) had a germline BRCA1/2 pathogenic variant and 1 patient (6.7%) had a somatic pathogenic variant in BRCA1. Among all of the carriers, 12 (80%) carried a BRCA1 mutation, including 7 frameshift insertion/deletion, 3 nonsense mutation, 1 exonic deletion, and 1 missense mutation; 3 (20%) had a BRCA2 mutation, including 1 frameshift insertion/deletion, 2 nonsense mutation (Supplementary Table 1). The BRCA1/2 carriers tended to be younger in age with a mean age of 46 years (range 37–63 years) compared to the non-carriers who had a mean age of 51 years (range 30–80 years) (*p* = 0.048, Table 1). In addition, the prevalence of BRCA1 mutations was 20.0% in the 55 TNBC patients who were diagnosed before the age of 60, and was only 6.7% in the 15 patients diagnosed at or above the age of 60 (Supplementary Table 2). BRCA1/2 mutations were also significantly associated with bilateral breast cancer (*p* = 0.043, Table 1). No significant differences were observed in histology, tumor size, tumor grade, lymph node involvement, pathological stage, or menopause at diagnosis.

**Table 1:** Basic clinicoathological parameters of the patients and their correlation with BRCA1/2 mutation status in breast cancers

Characteristics	All(n=70) (%)	Non-carriers(n=55) (%)	BRCA1/2 carrier(n=15) (%)	P
**Age**				
** Median (range)**	50 (30-80)	51 (30-80)	46 (37-63)	**0.048**
** ≤40 years**	15 (21.4)	11 (20.0)	4 (26.7)	0.723
** >40 years**	55 (78.6)	44 (80.0)	11 (73.3)	
**Menopause at diagnosis**				0.227
** Post-menopause**	33 (47.1)	28 (50.9)	5 (33.3)	
** Pre-menopause**	37 (52.9)	27 (49.1)	10 (66.7)	
**Histology**				0.577
** Ductal**	65 (92.9)	50 (90.9)	15 (100.0)	
** Other**	5 (7.1)	5 (9.1)	0 (0.0)	
**Tumor size (cm)**				0.319
** ≤2**	19	17 (30.9)	2 (13.3)	
** >2**	49	37 (67.3)	12 (80.0)	
** Unknow**	2	1 (1.8)	1 (6.7)	
**Lymph node metastasis**				0.386
** No**	44 (62.9)	33 (60.0)	11 (73.3)	
** Yes**	26 (37.1)	22 (40.0)	4 (26.7)	
**TNM stage**				0.888
** 0/I**	12 (17.1)	10 (18.2)	2 (13.3)	
** II**	48 (68.6)	37 (67.3)	11 (73.3)	
** III/IV**	10 (14.3)	8 (14.5)	2 (13.3)	
**Pathological stage**				1.0
** I/II**	8	6 (10.9)	2 (13.3)	
** III**	55	43 (78.2)	12 (80.0)	
** Unkown**	7	6 (10.9)	1 (6.6)	
**bilateral breast cancer**				**0.043**
** No**	68 (97.1)	55 (100.0)	13 (86.7)	
** Yes**	2 (2.9)	0 (0.0)	2 (13.3)	

### No predictive role of BRCA1/2 mutations was found for RFS or OS in TNBC patients

**Figure 1:**
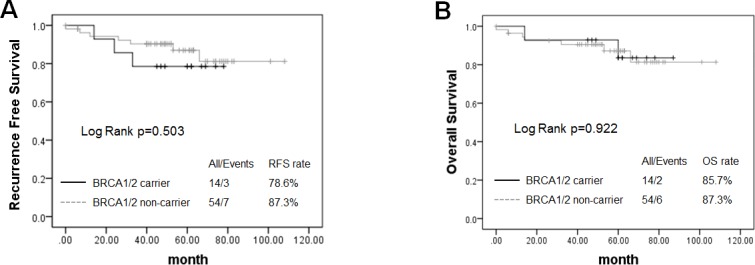
Kaplan–Meier survival plots showed that no predictive role of BRCA1/2 mutations was found for recurrence-free survival (RFS) **(A)** and overall survival (OS) **(B)** in TNBC patients.

Survival analysis was conducted among 68 patients with stage 0–III cancer, including 14 BRCA1/2 carriers and 54 non-carriers. The median follow-up was 54.5 months (range: 1–274 months). A total of 10 patients experienced local or distant metastases or died. The BRCA1/2 carriers shared similar short-term RFS and OS as non-carriers (RFS, log-rank *p* = 0.503; OS, log-rank *p* = 0.922) (Figure 1).

### Study selection for investigating the prognostic role of BRCA status by meta-analysis

**Figure 2:**
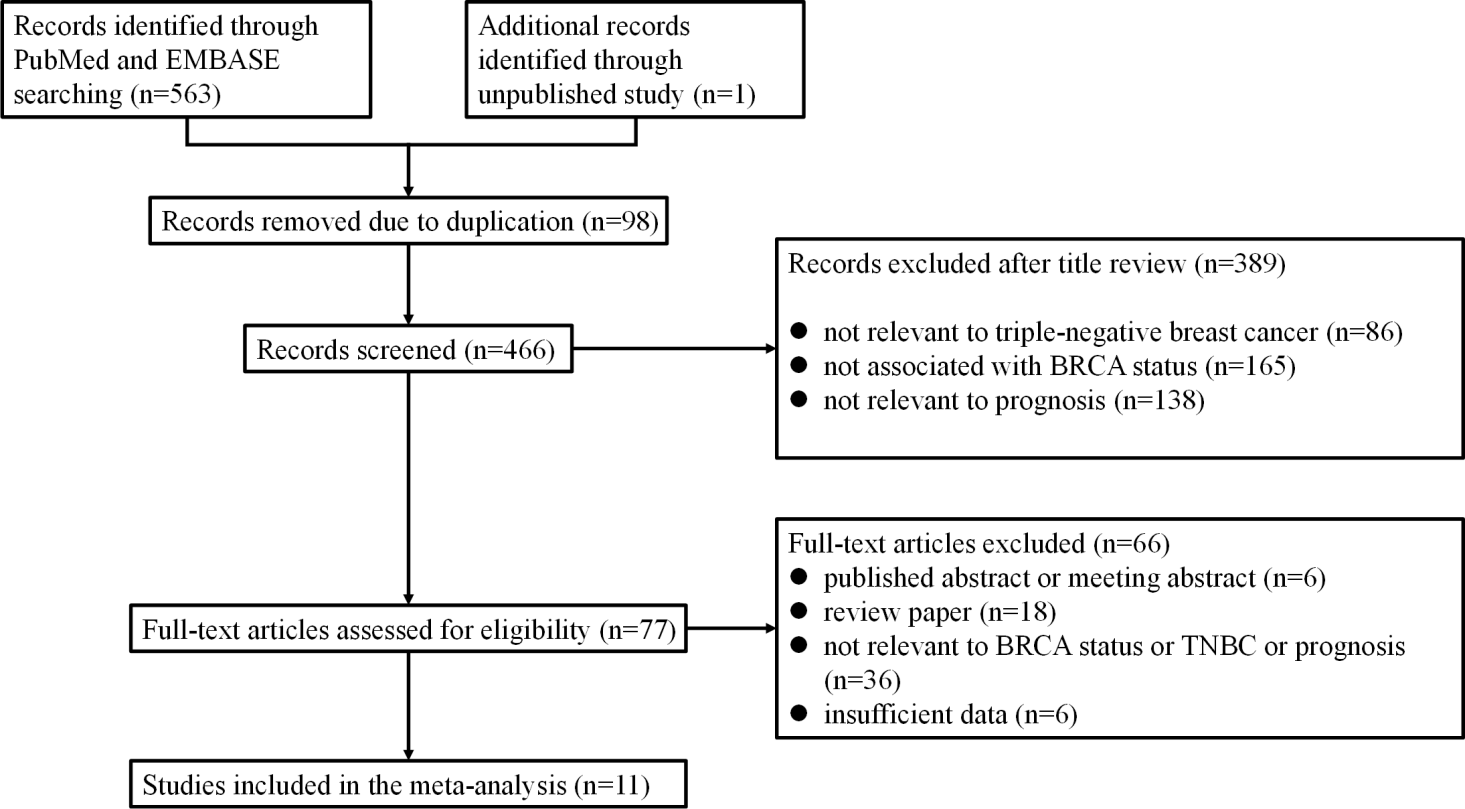
Flow chart of publication selection

The primary search yielded a total of 564 publications, 487 of which were excluded due to duplication or after screening the titles. The full text of the remaining 77 papers was reviewed, resulting in 66 being excluded and a total of 11 papers being used for the meta-analysis [18, 21–29] (Figure 2). All of the eligible studies were case-control studies. A total of six studies investigated BRCA1 promoter methylation, three studies investigated BRCA1/2 germline and/or somatic mutations, and two studies focused on low BRCA1 protein expression. As shown in Table 2, only studies on both BRCA1 and BRCA2 mutations were included in the meta-analysis. One study only investigated the germline BRCA1/2 mutation, whereas others investigated both germline and somatic BRCA1/2 mutations. Six studies analyzed BRCA1 promoter methylation, three of which were performed in patients with TNBC who received adjuvant chemotherapy. Additionally, methylation-specific PCR was a dominant testing method for detection of BRCA1 promoter methylation, except one study that used combined bisulfite and restriction analysis (Table 2).

**Table 2:** Characteristics of studies of triple-negative breast cancer patients with BRCA1/2 mutation, BRCA1 promoter methylation or low BRCA1 protein expression

BRCA status	First author	Study/published Year	Country	No.(cases/controls)	Age, median (range)	Tumor stage	Median/range follow-up (month)	Germline/somatic	Mutation types/detection	Cut off (%)
BRCA1/2 mutation	Bayraktar S [15]	1997-2010/2011	U.S.A	114/113	40 (21–74)	I-III	40.8/−	Germ	deleterious	-
	Gonzalez-Angulo AM[18]	1997-2006/2011	U.S.A	15/62	51 (27–83)	I-III	43/7–214	Germ+Somatic	deleterious	-
	Xie YX	2008-2014/−	China	14/54	50 (30-80)	I-III	31.5/1-274	Germ+Somatic	deleterious	-
BRCA1 methylation	Ignatov T [19]	2005-2008/2013	Germany	43/22	56 (34–87)	I-III	45/1–114	Somatic	-/MSP	-
	Xu Y [20]	1994-2002/2013	China	54/113	50 (25-87)	I-III	108/4.8–181.2	Somatic	-/MSP	-
	Sharma P [21]	1996-2008/2014	USA	11/26	52 (33-80)	I-III	64/8-148	Somatic	-/MSP	-
	Foedermayr M [26]	-/2014	Austria	10/14	47 (29-69)	I-III	27.5/−	Somatic	-/MSP	
	Yamashita N [22]	1990-2011/2015	Japan	11/51	57 (30-86)	I-III	120/−	Somatic	-/COBRA	-
	Zhu X [23]	1999-2008/2015	China	137/102	50 (25–83)	I-III	77.9/2.13–174	Somatic	-/MSP	-
BRCA1 downexpression	Chen L [25]	2001-2006,2015	China	107/43	51 (−)	I-III	96/−	Somatic	-/IHC	50
	Cho EY [24]	1997-2007,2011	Korea	-	73.5 (24.2–120.0)	IV	45/21–81	Somatic	-/IHC	SIs < 5

HR, homologous recombination; CIs, confidence intervals; MSP, methylation-specific PCR; COBRA, combined bisulfite and restriction analysis; IHC, immunohistochemistry; SIs, proportion scores.

Immunohistochemistry (IHC) was used in two studies to detect the expression of BRCA1 protein in TNBC, although the cut-off score for each study was different. In one study, immunostaining of less than 10% of tumor cells (with a cutoff value of 10% positive cells) was defined as low BRAC1 expression, whereas intensity × proportion scores < 5 were considered negative staining in another study (Table 2). Due to insufficient data, only studies on both BRCA1 and BRCA2 mutations were used for RFS analysis, and studies with only BRCA1 promoter methylation were included for DFS analysis.

### BRCA1 promoter methylation was a prognostic factor for worse OS in TNBC patients

**Figure 3:**
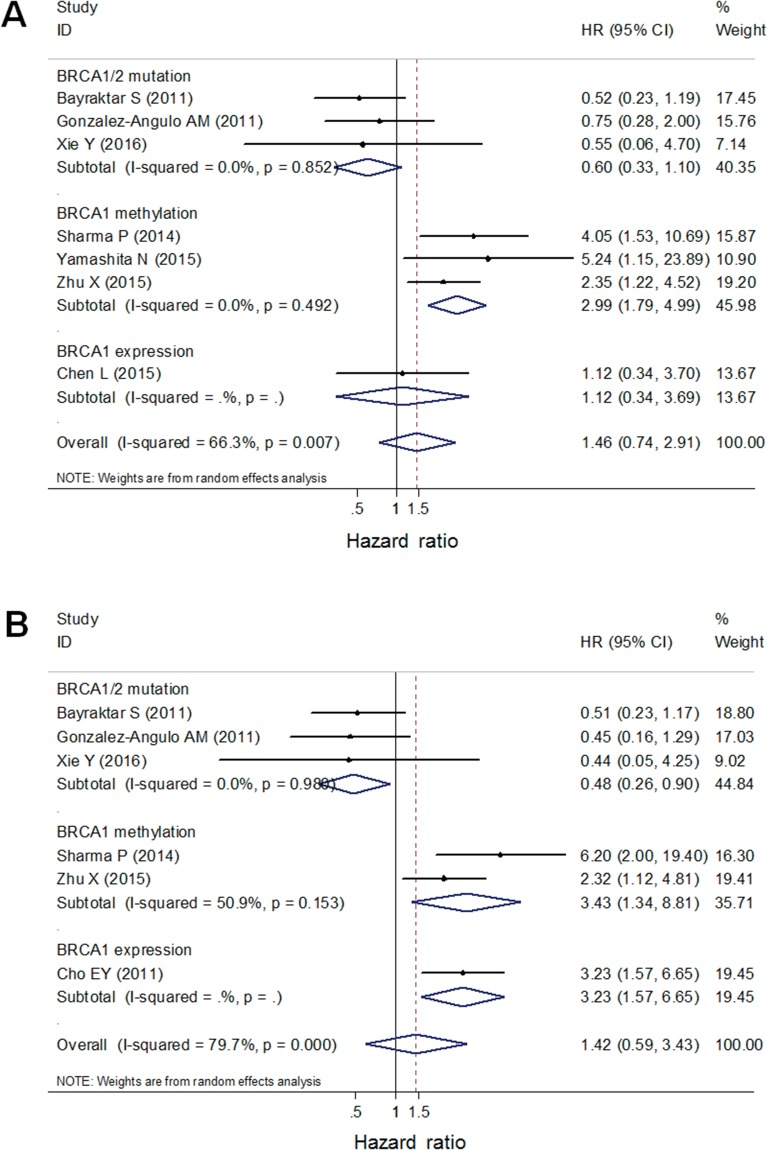
A forest plot showed that OS of TNBC patients was associated with BRCA1 promoter methylation, but not with BRCA1/2 germline/somatic mutations or low BRCA1 protein using univariate **(A)** and multivariate **(B)** analysis. Random-effect model was used for the analysis.

The significant heterogeneity between positive and negative trials enabled us to perform a quantitative aggregation of the survival data. We combined studies of germline and somatic BRCA1/2 mutations into one BRCA1/2 mutation subgroup in the meta-analysis. The overall meta-analysis for OS included three studies on BRCA1/2 mutations, three studies on BRCA1 promoter methylation, and two studies on low BRCA1 expression (Figure 3). Considering that the overall heterogeneity was significant (I^2^ = 66.3%, *p*=0.007 in the univariate analysis; I^2^ = 79.7%, *p* = 0.000 in the multivariate analysis), the random effects model was used and subgroup analysis was performed by considering BRCA mutation status. In general, BRCA dysfunction status was not associated with OS (hazard ratio [HR] = 1.46, 95% confidence interval [CI]: 0.74–2.91 in univariate analysis; HR = 1.42, 95% CI: 0.59–3.43 in multivariate analysis). However, in subgroup analyses, BRCA1 promoter methylation was a statistically significant prognostic factor for worse OS (HR = 2.99, 95% CI: 1.79–4.99 in univariate analysis; HR = 3.43, 95% CI: 1.34–8.81 in multivariate analysis) (Figure 3A, 3B). No statistically significant correlation was observed between OS and BRCA1/2 mutations or low BRCA1 expression (HR = 0.60, 95% CI: 0.33–1.10; HR = 1.12, 95% CI: 0.34–3.69, respectively, in univariate analysis; HR = 0.48, 95% CI: 0.26–0.90; HR = 3.23, 95% CI: 1.57–6.65, respectively, in multivariate analysis) (Figure 3A, 3B).

### BRCA1 promoter methylation was a predictor for longer DFS in TNBC patients

**Figure 4:**
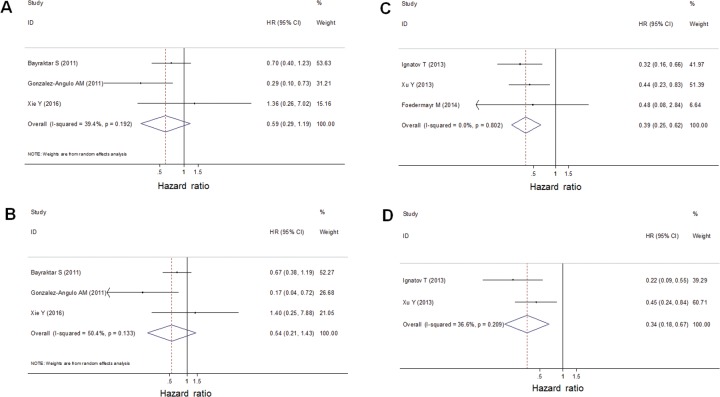
A forest plot showed that BRCA1/2 germline/somatic mutations were not associated with RFS of TNBC patients using univariate **(A)** and multivariate **(B)** analysis. Random-effect model was used for the analysis. A forest plot showed that BRCA1 promoter methylation was a predictor for longer DFS of TNBC using univariate **(C)** and multivariate **(D)** analysis. Fixed-effect model was used for the analysis.

The meta-analysis of BRCA1/2 mutations included three studies on RFS and that of BRCA1 promoter methylation included three studies on DFS. Heterogeneity of BRCA1/2 mutations was significant (I^2^ = 39.4%, *p* = 0.192 in univariate analysis; I^2^ = 50.4%, *p* = 0.133 in multivariate analysis). BRCA1/2 mutations were not associated with RFS (HR = 0.59, 95% CI: 0.29–1.19 in univariate analysis; HR = 0.54, 95% CI: 0.21–1.43 in multivariate analysis) (Figure 4A, 4B). However, the heterogeneity of BRCA1 promoter methylation was not significant (I^2^ = 0.0%, *p* = 0.802 in univariate analysis; I^2^ = 36.6%, *p* = 0.209 in multivariate analysis). Thus, the fixed effects model was used for the analysis. BRCA1 promoter methylation was a statistically significant predictor for longer DFS (HR = 0.39, 95% CI: 0.25–0.62 in univariate analysis; HR = 0.34, 95% CI: 0.18–0.67 in multivariate analysis) (Figure 4C, 4D).

### No publication bias was found for OS in the univariate or multivariate analysis

**Figure 5:**
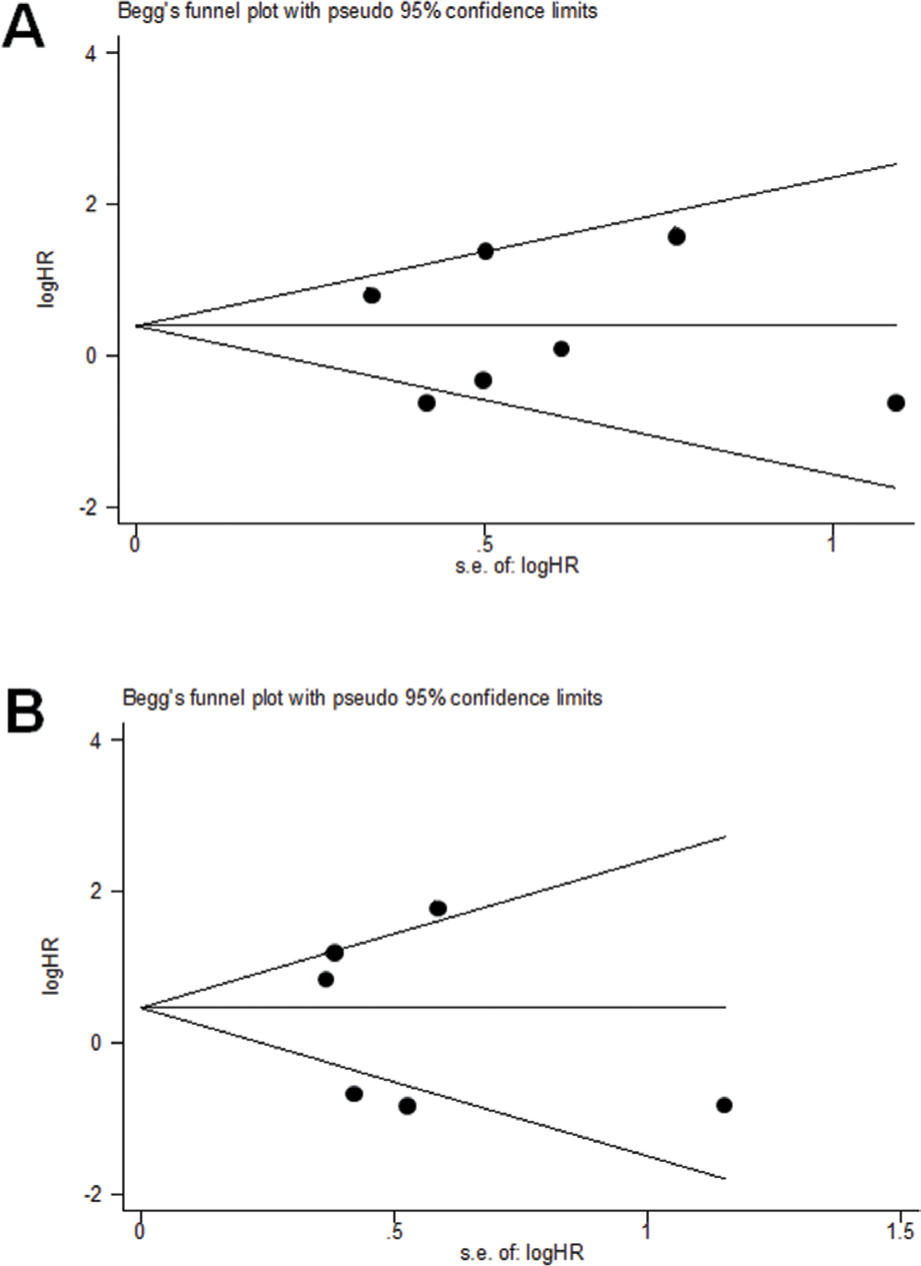
Egger's linear regression model and Begg's funnel plot showed that no publication bias was found for OS of TNBC in the univariate analysis **(A)**, or in the multivariate analysis **(B)**.

Publication bias statistics were determined using Egger's linear regression model and Begg's funnel plot. No publication bias was found for OS in the univariate analysis (Begg's test, *p* = 1.000; Egger's test *p* = 0.897) (Figure 5A), or in the multivariate analysis (Begg's test, *p* = 1.000; Egger's test *p* = 0.631) (Figure 5B).

## DISCUSSION

In this study, we explored the characteristics and prognostic implications of BRCA1/2 mutations in unselected Chinese TNBC patients. To the best of our knowledge, this is the first meta-analysis to investigate the survival of TNBC patients with BRCA1/2 germline/somatic mutations, BRCA1 promoter methylation, or low BRCA1 protein in one comprehensive report.

Our findings showed that 14 (20.0%) germline BRCA1/2 variant carriers occurred in 70 TNBC patients and occupied most of the BRCA1/2 mutations (14/15). These results are consistent with other reports, which confirmed that germline BRCA1/2 mutations are enriched in unselected TNBC patients (11.2–20.0%) [9, 30]. There are 15 unique pathogenic variants in this study. 7 (46.7%) are novel variants that were not reported before, including c.519del, c.2556_2557insTTCACTTTTC, c.2570T>A, c.4069_4070insTTGA, c.4712del, c.192T>G and c.6402_6406del. In addition, the BRCA1 mutation (c.981_982del) in the study is a founder mutation which has been previously reported in the Southern Chinese breast cancer patients [31]. The onset age of BRCA1/2 carriers was younger than that of non-carriers, supporting the fact that BRCA genes are a potential cancer risk factor. Furthermore, according to the National Comprehensive Cancer Network Guidelines (NCCN), TNBCs diagnosed in patients younger than 60 years of age can be considered for BRCA1 genetic testing. However, there is currently no criterion for genetic counseling in Chinese patients with TNBC. Our results support the criterion in NCCN guidelines. Although one study showed that the BRCA1 mutation frequency was higher in patients who were diagnosed at 50 years old or younger [16]. Nevertheless, more studies need to be performed in the future.

Studies and meta-analyses of breast cancer cases have shown that BRCA1 with or without BRCA2 mutations is linked to a poor outcome [14, 32]. Our findings in unselected TNBC patients demonstrated that BRCA1/2 mutations had no statistically significant correlation with short-term OS or RFS. This led us to question whether the molecular and pathological similarities between the BRCA1/2 mutant and TNBCs caused indistinctive outcomes between mutation carriers and non-carriers. However, the effect of BRCA dysfunction on the survival of TNBC patients remains controversial. To gain understanding on this issue, we performed a systematic review and meta-analysis to evaluate the prognostic role of BRCA1/2 dysfuction in TNBCs. Despite lack of evidence in the literature of better survival in BRCA1/2 mutation carriers, we explored the possibility that these mutations would confer an OS and RFS advantage. A previous study reported a better prognosis in BRCA1 mutation carriers compared to non-carriers in patients with sporadic breast cancer [33]. Furthermore, BRCA1 mutation carriers had increased breast cancer mortality if they did not receive chemotherapy [34]. Other studies have shown that TNBC patients with BRCA1 carriers are more likely to respond to neoadjuvant anthracycline-based regimens than non-carriers [16]. When treated with alkylating chemotherapy, similar survival rates were observed in BRCA1 mutation carriers and non-carriers in TNBC [35]. These findings suggest that deleterious BRCA1/2 mutations in TNBC do not have negative prognostic significance. In fact, it is more likely that patients with triple-negative BRCA mutants may be more sensitive to chemotherapy than those with other high-grade TNBCs.

It has been hypothesized that promoter methylation may be a main epigenetic mechanism underlying inactivation of the BRCA1 gene in sporadic breast cancer [36]. BRCA1-methylated sporadic breast cancers tend to be ‘BRCA-like’ in that they have a triple-negative phenotype [37]. Furthermore, it was observed that BRCA1 methylation more often occurs in TNBC [36, 38]. Our results showed that BRCA1 promoter methylation was a poor prognostic factor for OS in TNBC patients, regardless of whether they received chemotherapy. These data were in accordance with previous findings which showed that BRCA1 methylation was significantly correlated with poor OS in sporadic breast cancers [15].

It is worth noting that BRCA1 methylation was associated with favorable DFS in TNBC patients who received adjuvant chemotherapy. The results indicate that BRCA1 promoter methylation in TNBC may be predictive of chemo-sensitivity. However, studies on the role of BRCA2 methylation on the prognosis of patients with TNBC are limited.

Because the analysis of the prognostic role of low BRCA1 expression in TNBCs was done from only two papers, we are still unable to make definitive conclusions. Thus, additional studies are needed in larger cohorts. Due to the lack of targeted or endocrine therapy for TNBC, chemotherapy is still the main treatment. TNBC patients with BRCA1 mutations or promoter methylation may be more sensitive to chemotherapy, and thus may benefit from adjuvant chemotherapy [4, 39, 40]. Furthermore, multiple clinical trials on the efficacy of chemotherapy with PARP inhibitors in metastatic TNBC patients have shown positive results [41, 42]. Thus, it is vital to understand the role of BRCA1/2 dysfunction in TNBC. BRCA1/2 gene abnormality could lead to a defect in the HR pathway of DNA repair. The Fanconi anaemia (FA)/BRCA pathway is such an important pathway involved in the damaged DNA repair and maintenance of chromosomal stability [43]. The mechanisms by which FA proteins and BRCA proteins and their role in TNBC should be investigated. Loss of CDK1 activity, which maintains BRCA1 protein stability, may occur in TNBC of BRCA1/2 gene abnormality [44]. In addition, loss of RAD51 expression, a necessary recombinase in the HR complex that associates with BRCA1/2, also needs to be validation in the future study [45].

There were several limitations in this study. The number of studies eligible for the meta-analysis was small. In addition, heterogeneity existed regarding the patient selection criteria, chemotherapy regimens, and follow-up period. Therefore, more studies are needed to investigate the effect of BRCA1/2 dysfunction on the prognosis of TNBC patients.

## MATERIALS AND METHODS

### Patient population and data collection

Ethical approval for this project was obtained from the Clinical Test and Biomedical Ethics Committee of West China Hospital, Sichuan University. Written informed consent was provided by all the patients. All methods were carried out in accordance with the approved guidelines.

A total of 5103 patients with primary breast cancer were registered in the Breast Cancer Information Management System (BCIMS) at West China Hospital, Sichuan University between February 2008 and February 2014. Among the registered patients, 4791 were recruited who had undergone surgery in the Department of Thyroid and Breast Surgery, regardless of age at diagnosis or family history of breast cancer. Finally, 70 patients were eligible for the BRCA test after excluding those who failed follow-up, lacked complete clinical information, or were unable to provide a sufficient amount of tumor tissue, matched frozen distal adjacent normal tissue, or peripheral blood.

### Pathologic assessment and mutation analysis

Clinical and pathological characteristics were extracted from the BCIMS. IHC scoring for ER and PR was performed according to the Guidelines for Testing of ER and PR in Breast Cancer [46]. IHC and fluorescence *in situ* hybridization scoring for HER-2 was conducted following the Guidelines for HER-2 Detection in Breast Cancer [47]. Standard therapy was defined as administration of comprehensive therapy according to NCCN and St. Gallen International Expert Consensus [48].

BRCA testing was performed using germline DNA (from blood) and somatic DNA (from tumor tissue). Details of the comprehensive NGS workflow for testing and analyzing tumor BRCA1/2 variants were described in our previous work [49]. Germline mutation were interpreted according to the American College of Medical Genetics and Genomics (ACMG) and other studies [50]. Briefly, variants that produce premature termination codons which are associated with non-functional or truncated proteins were classified as pathogenic (P) variants in our study: such as nonsense mutations, frameshift mutations, splice site mutations and exonic deletions. Similarly, inactivating somatic variants were considered as pathogenic variants: such as nonsense mutations and frameshift mutations [49]. The results were categorized as either positive or negative for a deleterious mutation.

### Literature search strategy

For the meta-analysis, a systematic literature search of PubMed and Embase databases (last search updated in May 2016) was conducted to identify papers that evaluated the effect of BRCA1/2 status on the survival of patients with TNBC. The following search terms were used: “BRCA1”, “BRCA2” or “BRCA1/2” and “triple-negative breast cancer” or “triple negative breast cancer” and “prognosis” or “prognostic” or “survival” or “outcome”. In addition, a manual search for other relevant articles was carried out using the reference lists of eligible studies.

### Selection criteria

Eligible studies met the following predefined criteria: (1) Case-control studies that addressed the prognosis of TNBC patients, according to BRCA status (BRCA1/2 mutations, BRCA1 promoter methylation in the primary tumor, and/or BRCA1/2 protein expression); (2) Studies in which the primary outcome was OS, DFS and/or RFS; (3) Papers with sufficient published data for calculating the hazard ratios (HRs) and 95% confidence intervals (CIs); (4) Studies that were confined to human females. (5) The studies with the largest sample size were included if the same patient population were overlapped among publications.

### Data extraction and quality assessment

The following data were extracted from all included studies: the first author's name, year of study or publication, country, sample size, patient age, tumor stage, follow-up period, origin of BRCA status, detection method, and cut-off level. Quality assessment of the primary studies was executed using the Newcastle-Ottawa Quality Assessment Scale [51].

### Statistical analysis

Quantitative data analysis was performed with the two-tailed Student's *t*-test, one-way analysis of variance followed by Dunnett's multiple comparison post-test. Kaplan–Meier and log-rank analyses were used to assess the survival between subgroups. A Cox proportional hazards model was used to determine the independent factors of survival and recurrence based on the variables selected in univariate and multivariate analyses. HR was invoked as a measure of the prognostic value. HR > 1 indicated poor survival for the group with BRCA dysfunction status, whereas HR < 1 indicated a favorable prognosis. A Cochrane's Q test was implemented to test heterogeneity among studies. The p value of the Q test was < 0.1, which suggested the presence of heterogeneity, and the random effects model (DerSimonian-Laird method)[52] was used to calculate pooled HRs. Otherwise, heterogeneity was absent and the fixed effects model (Mantel-Haenszel method) [53] was more appropriate. In addition, the degree of heterogeneity was assessed by the I^2^-test. The value of *I^2^* ranged from 0% to 100% and was generally considered no heterogeneity for I^2^=0, moderate heterogeneity for 25%, large heterogeneity to 50%, and extreme heterogeneity for 75% [54]. Furthermore, a funnel plot and test with Begg and Egger's tests [55–57] were utilized to investigate any possible publication bias. The funnel plot was visually symmetrical and the P value of Begger or Egger's test was greater than 0.05, which indicated that there was no statistically significant publication bias. All statistical tests were two-tailed and P values less than 0.05 were considered statistically significant. Statistical analyses were done with SPSS 20.0 (SPSS Inc., Chicago, IL, USA), STATA software version 11.0 and Review Manager 5.3 software.

## SUPPLEMENTARY TABLES


