# Supplementary material for: The role of BRCA status on prognosis in patients with triple-negative breast cancer
Source: Oncotarget. 2017 Aug 3;8(50):87151–62. doi: 10.18632/oncotarget.19895 (PMC5675622; doi:10.18632/oncotarget.19895)
Supplement: Supplementary file 1 [file oncotarget-08-87151-s001.pdf]

The role of BRCA status on prognosis in patients with triple-negative breast cancer

SUPPLEMENTARY MATERIALS

Supplementary Table 1: Detailed information about *BRCA1* and *BRCA2* mutations

Gene	Exon	Nucleotide change	Amino acid change	Category[a]	Variant type	Somatic/ germline
BRCA1	2	c.66dup	E23INS	P	Frameshift insertion/ deletion	Germline
BRCA1	8	c.519del	Q174DEL	P	Frameshift insertion/ deletion	Germline
BRCA1	11	c.981_982del	C328DEL	P	Frameshift insertion/ deletion	Germline
BRCA1	11	c.2556_2557insTTCACTTTTC	L852INS	P	Frameshift insertion/ deletion	Germline
BRCA1	11	c.2570T>A	L857*	P	Nonsense mutation	Germline
BRCA1	11	c.4069_4070insTTGA	Q1356DEL	P	Frameshift insertion/ deletion	Germline
BRCA1	16	c.4801A>T	K1601*	P	Nonsense mutation	Germline
BRCA1	16	c.4712del	F1571DEL	P	Frameshift insertion/ deletion	Germline
BRCA1	24	c.5470_5477del	I1824DEL	P	Frameshift insertion/ deletion	Germline
BRCA1	24	c.5503C>T	R1835*	P	Nonsense mutation	Somatic
BRCA1	16-17	c.4676_5074del	E1559_T1691del	P	Exonic deletion	Germline
BRCA1	5	c.192T>G	C64W	P	Missense mutation	Germline
BRCA2	10	c.1399A>T	K467*	P	Nonsense mutation	Germline
BRCA2	11	c.6402_6406del	N2134DEL	P	Frameshift insertion/ deletion	Germline
BRCA2	11	c.5645C>A	S1882*	P	Nonsense mutation	Germline

[a]Variants were annotated using Alamut software (Interactive biosoftware, France) integrated with multiple databases. Germline variants were interpreted according to the American College of Medical Genetics and Genomics (ACMG). Variants that produce premature termination codons which are associated with non-functional or truncated proteins were classified as pathogenic (P) variants: such as nonsense mutations, frameshift mutations, splice site mutations and exonic deletions. Similarly, inactivating somatic variants were considered as pathogenic variants: such as nonsense mutations and frameshift mutations.

Supplementary Table 2: Age distribution of BRCA1 mutation in 70 triple-negative breast cancer patients

Age of onset	Total TNBC No. of patients	BRCA1 carrier	
		No.	%
**<40**	10	1	10
**<50**	38	8	21.1
**<60**	55	11	20.0
**<70**	67	12	17.9
**≤80**	70	12	17.1
